# Sequential association of myogenic regulatory factors and E proteins at muscle-specific genes

**DOI:** 10.1186/2044-5040-1-14

**Published:** 2011-04-04

**Authors:** Priya Londhe, Judith K Davie

**Affiliations:** 1 Department of Biochemistry and Molecular Biology, Southern Illinois University School of Medicine, 1245 Lincoln Drive, Carbondale, IL 62901, USA

## Abstract

**Background:**

Gene expression in skeletal muscle is controlled by a family of basic helix-loop-helix transcription factors known as the myogenic regulatory factors (MRFs). The MRFs work in conjunction with E proteins to regulate gene expression during myogenesis. However, the precise mechanism by which the MRFs activate gene expression is unclear. In this work, we sought to define the binding profiles of MRFs and E proteins on muscle-specific genes throughout a time course of differentiation.

**Results:**

We performed chromatin immunoprecipitation (ChIP) assays for myogenin, MyoD, Myf5 and E proteins over a time course of C_2_C_12 _differentiation, resulting in several surprising findings. The pattern of recruitment is specific to each promoter tested. The recruitment of E proteins often coincides with the arrival of the MRFs, but the binding profile does not entirely overlap with the MRF binding profiles. We found that E12/E47 is bound to certain promoters during proliferation, but every gene tested is preferentially bound by HEB during differentiation. We also show that MyoD, myogenin and Myf5 have transient roles on each of these promoters during muscle differentiation. We also found that RNA polymerase II occupancy correlates with the transcription profile of these promoters. ChIP sequencing assays confirmed that MyoD, myogenin and Myf5 co-occupy promoters.

**Conclusions:**

Our data reveal the sequential association of MyoD, myogenin, Myf5 and HEB on muscle-specific promoters. These data suggest that each of the MRFs, including Myf5, contribute to gene expression at each of the geness analyzed here.. The dynamic binding profiles observed suggest that MRFs and E proteins are recruited independently to promoters.

## Background

The entire process of skeletal muscle differentiation is controlled by four highly related basic helix-loop-helix (bHLH) proteins referred to as the myogenic regulatory factors (MRFs). The MRFs have distinct but overlapping patterns of gene expression during muscle development [1]. Gene knockouts of each factor in the mouse have revealed that each MRF has a unique role in skeletal muscle differentiation. Myf5, Myf6 (also known as MRF4) and MyoD are not required for viability, although each mutant has a distinct phenotype [2]. In the combined absence of Myf5, Myf6 and MyoD, myoblasts are not specified and no skeletal muscle forms, resulting in a lethal phenotype [3]. Myogenin is the only MRF singly required for viability [4,5]. Mice heterozygous for the null allele appear normal, while mice lacking *myogenin *die at birth. The *myogenin*-null mice have myoblasts, but very few muscle fibers. This suggests that *myogenin *is not required for the specification of skeletal muscle, but is required for the later stages of myofiber fusion.

MyoD and myogenin have been shown to bind highly overlapping gene sets, although certain genes appear to be selective for either factor [6,7]. However, the high degree of overlap in the binding patterns suggests that the majority of genes utilize both factors to activate gene expression. Previous work has shown that certain genes require the sequential expression of both MyoD and myogenin to activate gene expression [7]. The present work suggests that the activation of specific targets requires MyoD and its associated chromatin-modifying activities before myogenin can activate transcription. Why MyoD cannot activate transcription without myogenin on these genes is still unknown.

Recent work on Myf5 has revealed unexpected roles for this factor in adult animals. As mentioned above, *Myf5 *functions as a determination gene in early myogenesis. The role of Myf5 in later stages is unclear. In the absence of MyoD, Myf6 or myogenin, Myf5 is unable to promote differentiation from myoblasts [8]. This finding suggests that Myf5 functions only in muscle progenitor cells (MPCs) and myoblasts. However, recent work has shown that *Myf5*-null mice exhibit impaired muscle regeneration with a significant increase in muscle fiber hypertrophy and a delay in differentiation [9]. However, satellite cell numbers were not significantly altered in the *Myf5*-null animals, although a modest impaired proliferation was observed under some conditions *in vitro*. This work highlights the questions still remaining about the roles of the MRFs at distinct stages in myogenesis.

All bHLH transcription factors function as either homodimers or heterodimers. The bHLH transcription factors are loosely grouped into several categories: the widely expressed E proteins, including the *E2A *gene products E12 and E47, HEB, E2-2 and Daughterless, are in the class I category and the MRF family is included in the tissue-specific class II category. Class II bHLH proteins form weak homodimers and preferentially heterodimerize with E proteins [10]. Prior *in vitro *experiments have demonstrated that the class II MRFs form avid heterodimers with class I E proteins, but homodimerize poorly in the presence of DNA sites [11-14]. Thus, it is thought that the MRFs function as heterodimers with ubiquitous E proteins. The E proteins suggested to be involved in skeletal muscle differentiation are the *E2A *gene products E12 and E47, as well as HEB. Recent work has suggested that HEB may be the primary E protein that regulates skeletal muscle differentiation [15].

The MRFs all bind the canonical E-box consensus sequence, CANNTG. Genome-wide binding studies have revealed that both MyoD and myogenin preferentially bind E boxes with a consensus sequence of CASCTG (International Union of Pure and Applied Chemistry nomenclature http://www.iupac.org), where S represents G or C [7,16]. The site recognized at the highest frequency is CAGCTG. The sequences flanking the E box also make important contributions to the binding affinity and contribute to the overall consensus sequence elements determined for MyoD and myogenin [11,16].

Given the high degree of overlap detected in the genome occupancy of MyoD and myogenin, we were interested in understanding the binding profile of these factors over a time course of differentiation. We were also interested in the binding profile of Myf5, as binding data for this factor during differentiation have not been reported. We also sought to compare the DNA-bound profiles of the MRFs with the E proteins. Thus, we initiated a temporal analysis of the binding of MyoD, Myf5, myogenin and the E proteins in C_2_C_12 _cells, a widely used cell culture model for myoblast differentiation. These binding profiles were correlated with the levels of mRNA present in the cells, the levels of RNA polymerase II (RNAP II) occupancy and histone H3 acetylation present at the promoters. We show several novel findings. Surprisingly, we have found that the pattern of recruitment is unique to each gene, although some common features arise. As others have observed, we saw an early association of MyoD with most of these genes. In a cooperative pattern, myogenin then binds many of these promoters following MyoD. Unexpectedly, we found that Myf5 is also associated with genes expressed late in differentiation and often colocalizes with myogenin. We show that this colocalization also occurs *in vivo *at a late embryonic time point. The binding of each of the MRFs is transient. We also show that the occupancy of the E proteins is transient and that the occupancy often peaks concurrently with the peak in gene transcription. While the *E2A *gene products could be detected on a few genes in our study in proliferating cells, HEB does appear to be the dominant E protein used during differentiation. At the genes occupied by E12/E47 in early myogenesis, we detected a switch in occupancy for HEB during differentiation. Taken together, our data suggest new models for the recruitment of MRFs and E proteins and support a novel role for Myf5 during differentiation.

## Results

### Time course of MRF and E protein expression

**Figure 1:**
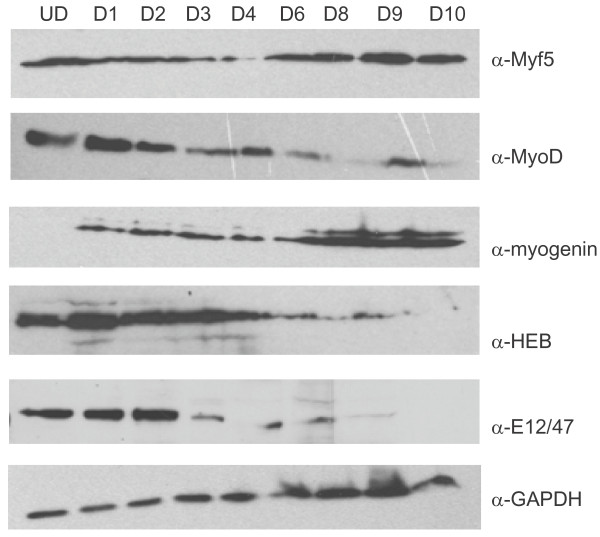
**Expression of myogenic regulatory factors (MRFs) and E proteins over a time course of differentiation in C_2_C_12 _cells**. C_2_C_12 _cells were differentiated for the indicated number of days and harvested for protein (UD, undifferentiated cells; D1-D10, cells differentiated for the indicated number of days). Protein concentration was determined for each extract and used to normalize the sample loading. Parallel blots were probed for each of the indicated antibodies as described in Methods.

To initiate this work, we first characterized the available antibodies for these studies and confirmed MRF and E protein expression patterns over a time course of differentiation. We tested well-characterized antibodies for MyoD and myogenin [17,18]. We found that antibodies against MyoD and myogenin immunoprecipitated the target protein (data not shown) and did not recognize any of the other MRFs (Additional file 1 Figure S1). For Myf5, we tested commercially available antibodies for their ability to recognize and immunoprecipitate Myf5 specifically. We identified one antibody that immunoprecipitated Myf5 exclusively (Additional file 1 Figure S2), and this antibody was used for all the studies presented. We did note that the antibody does recognize recombinant MyoD by using Western blot analysis, but we could not immunoprecipitate this protein (Additional file 1 Figure S2). For the E proteins, we used antibodies raised against HEB and the *E2A *gene products E12 and E47. The HEB antibody recognized E12/E47 on the basis of Western blot analysis, but immunoprecipitated HEB specifically (Additional file 1 Figure S3). The antibody against E12/E47 did not recognize or immunoprecipitate recombinant HEB (Additional file 1 Figure S4). Next, we examined the expression profile of the MRFs and HEB over an extended time course of C_2_C_12 _differentiation (Figure 1). As previously observed, MyoD and Myf5 were expressed in proliferating myoblasts. MyoD levels increased upon differentiation, but then rapidly decreased after two days of differentiation. Unexpectedly, Myf5 was expressed throughout the entire time course. Myogenin was not detectable in proliferating cells, but was rapidly upregulated upon differentiation. HEB was also expressed in proliferating myoblasts, but was steadily downregulated after about four days of differentiation. The E12/E47 proteins were also expressed in proliferating myoblasts, but the expression decreased after two days of differentiation.

### Expression of genes chosen for analysis

Several genes were chosen for this analysis. We chose *muscle creatine kinase (Ckm) *and *desmin (Des)*, as both are well-characterized genes whose expression increases during differentiation. As the regulatory regions of these genes have been studied extensively by others, promoter proximal binding sites for the MRFs are well defined [19-25]. We also chose the *fast-twitch skeletal muscle troponin I, type 2 *(*Tnni2*) and *leiomodin 2 *(*Lmod2*) genes, as these have been characterized as myogenin-dependent targets in embryonic skeletal muscle during embryogenesis, and the promoter proximal MRF binding sites are known [26]. We also chose *titin cap *(*Tcap*), also known as *telethonin*. We have recently characterized the promoter proximal regulatory elements of the gene encoding Tcap and identified a promoter proximal fragment that recapitulates the expression pattern of *Tcap *in reporter assays and is bound by myogenin *in vivo *[27]. For each of these genes, we profiled the change in RNA expression profiles over a time course of differentiation.

**Figure 2:**
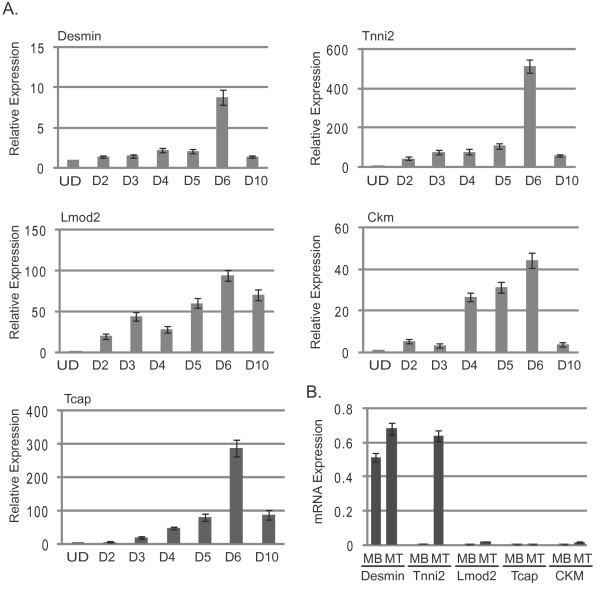
**Expression profiles of genes upregulated during skeletal muscle differentiation in C_2_C_12 _cells**. **(A) **Expression profiles of individual gene expression. Shown are graphs representing the change in expression of each gene examined in this study observed at each day of differentiation with respect to the expression level detected in proliferating C_2_C_12 _myoblast cells. **(B) **The relative transcription levels of each of the genes in this study under conditions of proliferation or differentiation are shown. Expression of each gene was determined by quantitative real-time polymerase chain reaction (qRT-PCR) assay and normalized to *HPRT *levels. Numbers indicate the fold change between undifferentiated samples (MB, myoblasts) and samples differentiated for six days (MT, myotubes). **(A and B) **Calculations of the relative fold changes in gene expression and mRNA expression are described in Methods. qRT-PCR assays were performed in triplicate on cDNA samples derived from independent RNA isolations. All data are normalized to the expression level of *HPRT*. Error bars represent standard deviations of the mean.

For each gene, we saw that expression increased when cells began to differentiate, as expected (Figure 2A). Unexpectedly, we also observed that the expression continued to increase over several days of differentiationand reached very high levels of expression after six days of differentiation. We also observed that the expression levels significantly decreased for each gene, with the exception of *Lmod2*, after ten days in differentiation conditions. We also wanted to compare the relative expression levels of the chosen gene set. To perform this analysis, we compared the expression of each gene to a constitutive housekeeping gene, *HPRT*, in both proliferating myoblasts and differentiated myotubes (Figure 2B). This analysis revealed that *Des *was expressed at a much higher level in proliferating myoblasts than any of the other genes examined in this study. We also observed that *Tnni2 *and *Des *were expressed at approximately the same high level in differentiated cells. The expression levels of *Lmod2*, *Tcap *and *Ckm *increased significantly but did not reach the level of activation of *Tnni2 *or *Des *in this time course.

### Binding of MRFs and E proteins to muscle-specific genes

**Figure 3:**
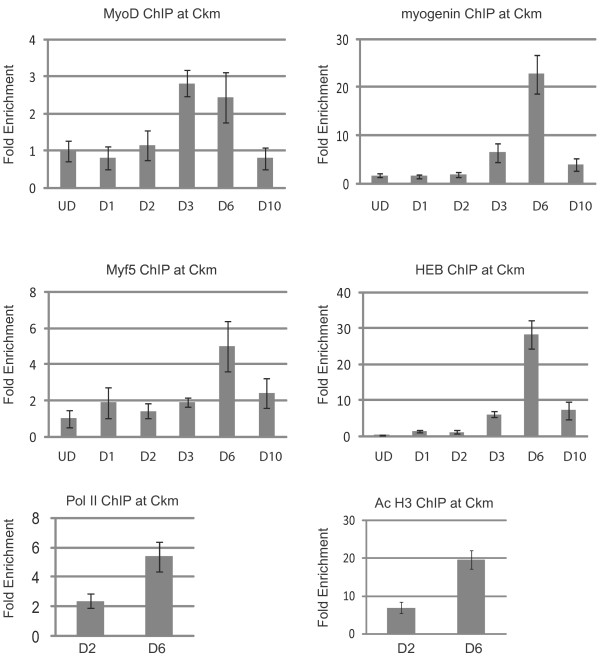
**Chromatin immunoprecipitation (ChIP) analysis of the *muscle creatine kinase *(*Ckm*) promoter**. Cross-linked extracts from proliferating myoblasts (UD, undifferentiated cells) and myofibers in differentiation media for the indicated number of days (D1-D10) were immunoprecipitated with antibodies against myogenin, MyoD, Myf5, HEB, RNA polymerase II (RNAP II), histone H3 acetylated at lysine 9 and/or 18 (H3 Ac9*/*18) or IgG. Immunoprecipitated DNA was purified and amplified with primers specific to the promoter of *Ckm*. Relative enrichment at the *IgH *locus was used to normalize the data. The fold enrichment values were calculated as described in Methods.

Next, we profiled the binding of MyoD, myogenin, Myf5 and E proteins over the time course of differentiation on each of these gene promoters. Proliferating cells and cells differentiated for one, two, three, six and ten days were used for the analysis. Surprisingly, we found that the pattern of recruitment was specific to each gene tested. First, we examined the well-characterized *Ckm *promoter [20-22]. For this analysis, we chose to examine the enhancer 1 element located upstream of the first noncoding exon of *Ckm *that contains one E box with the sequence CAGCTG, the preferred binding site for MyoD and myogenin. In accordance with recent chromatin immunoprecipitation sequencing (ChIP-seq) studies for MyoD [16], we did not detect MyoD at the *Ckm *enhancer in proliferating myoblasts (Figure 3). We also did not detect myogenin, Myf5 or HEB at this time point. After three days of differentiation, MyoD, myogenin and HEB were detectable at the *Ckm *enhancer. However, six days after differentiation, we observed greatly enriched binding of HEB and myogenin compared to the relatively unchanged binding of MyoD. We also detected Myf5 at this enhancer at this time point. By ten days of differentiation, the MRFs and HEB appeared to be departing the promoter. While the binding was significantly enriched over background for all factors except MyoD, the levels at ten days of differentiation were greatly reduced from those observed at six days of differentiation. The dynamic profiles of the MRFs and E proteins were surprising to us, so we also compared the levels of RNAP II occupancy as a measure of transcriptional activity. As histone H3 acetylation is also a marker of active genes and increases in histone H3 acetylation are correlated with MyoD binding, we also examined the level of histone H3 acetylation at these promoters. For these assays, we compared the binding profile of RNAP II and acetylated histone H3 (AcH3) at two days of differentiation and at six days of differentiation. We found that both RNAP II binding and histone H3 acetylation increased at six days of differentiation, consistent with the transcriptional profiling and the increased occupancy of all the MRFs and HEB.

**Figure 4:**
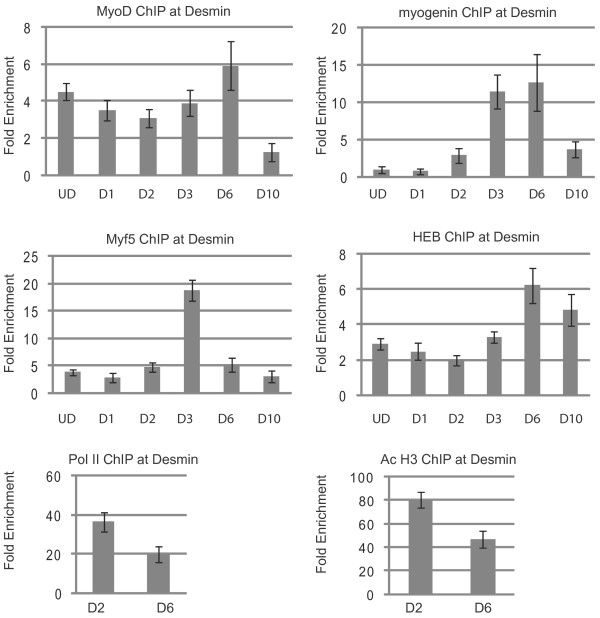
**Chromatin immunoprecipitation (ChIP) analysis of the *desmin *(*Des*) promoter**. Cross-linked extracts from proliferating myoblasts (UD, undifferentiated cells) and myofibers in differentiation media for the indicated number of days (D1-D10) were immunoprecipitated with antibodies against myogenin, MyoD, Myf5, HEB, RNA polymerase II (RNAP II), histone H3 acetylated at lysine 9 and/or 18 (H3 Ac9*/*18) or IgG. Immunoprecipitated DNA was purified and amplified with primers specific to the promoter of *Des*. Relative enrichment at the *IgH *locus was used to normalize the data. The fold enrichment values were calculated as described in Methods.

At *Des*, the most robustly expressed gene examined in this study, we examined the previously characterized promoter proximal enhancer element [24]. This region immediately upstream of the transcriptional start site contains one E box with the sequence CAGCTG. We observed that MyoD bound to the *Des *promoter in proliferating cells, again consistent with the recent ChIP-seq study (Figure 4). MyoD remained bound to the promoter as the cells differentiated, with the binding ratio peaking at six days of differentiation. Myf5 and HEB were also present on the *Des *promoter in proliferating cells. A peak of Myf5 binding was observed after three days of differentiation, when a peak of myogenin binding was also detected. By six days of differentiation, Myf5 binding was still detectable, but at greatly reduced levels. The levels continued to decrease after ten days of differentiation. The level of HEB remained fairly constant during the first two days of differentiation, but began to increase after three days and steadily increased until six days of differentiation. HEB remained bound to the promoter after ten days of differentiation. Myogenin was not detected at the promoter in proliferating cells. Myogenin was detectable at the promoter after two days of differentiation, and its levels were greatly increased after three days of differentiation. The levels remained high at six days of differentiation. After ten days of differentiation, HEB levels remained relatively unchanged, but the binding ratios of the MRFs were greatly decreased. MyoD was no longer detected at the promoter, and only low levels of both myogenin and Myf5 remained associated with the promoter. We observed that RNAP II and AcH3 were associated with the promoter after two days of differentiation, and, surprisingly, those levels moderately decreased after six days of differentiation. We note that the relative fold enrichment of RNAP II and AcH3 was very high at *Des*, the most highly expressed gene in this study. This indicates that the number of *Des *promoters bound by RNAP II was much higher than what was observed at other promoters.

**Figure 5:**
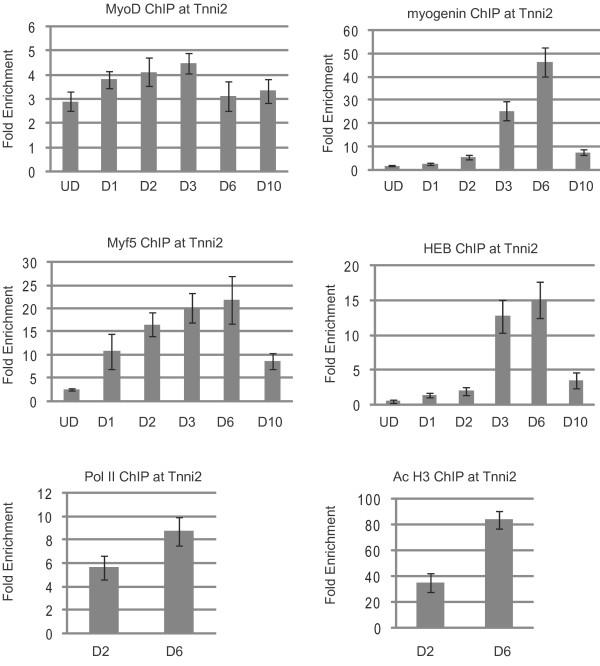
**Chromatin immunoprecipitation (ChIP) analysis of *fast-twitch skeletal muscle troponin I, type 2 *(*Tnni2*)**. Cross-linked extracts from proliferating myoblasts (UD, undifferentiated cells) and myofibers in differentiation media for the indicated number of days (D1-D10) were immunoprecipitated with antibodies against myogenin, MyoD, Myf5, HEB, RNA polymerase II (RNAP II), histone H3 acetylated at lysine 9 and/or 18 (H3Ac9*/*18) or IgG. Immunoprecipitated DNA was purified and amplified with primers specific to the promoter of *Tnni2*. Relative enrichments at the *IgH *locus were used to normalize the data. The fold enrichment values were calculated as described in Methods.

We next examined the *Tnni2 *promoter, which, like *Des*, is highly expressed in differentiating C_2_C_12 _cells (Figure 5). Previous work has shown that the expression of *Tnni2 *is highly dependent on myogenin *in vivo *[26]. The regulatory elements of *Tnni2 *are uncharacterized, so we chose to analyze a highly conserved noncoding sequence immediately upstream of the transcriptional start site. The transcriptional start site of *Tnni2 *is predicted to encode a short 5' untranslated region (5' UTR) that initiates 23 bp prior to the translational start site. The conserved noncoding region is approximately 300 bp upstream of the start of transcription and contains two E boxes. The sequence of the promoter distal E box is CACCTG, while the sequence of the promoter proximal E box is CAGCTG. The E boxes are separated by only 35 bp, so binding to either box could not be distinguished in our assay. As was true of *Des*, we observed an association of MyoD with the *Tnni2 *promoter in proliferating cells. The levels remained relatively unchanged during the time course of differentiation, although small variations were observed. Myf5 was recruited to the promoter upon the first day of differentiation, and the levels continued to increase until after day six. By day ten of differentiation, Myf5 levels were greatly reduced. The recruitment of HEB was particularly surprising at this promoter. Here, we saw that HEB was not recruited to the promoter before two days of differentiation. The levels continued to increase at six days of differentiation, but binding rapidly declined after this point. At this promoter, the binding pattern of HEB completely overlapped with the binding pattern of myogenin. Myogenin could be detected at the promoter at two days of differentiation, but binding was greatly enhanced after three days of differentiation. The levels continued to increase at six days of differentiation, but rapidly decreased after this point. At *Tnni2*, we observed that while RNAP II and AcH3 were present at two days of differentiation, the levels significantly increased at six days of differentiation. After six days of differentiation, AcH3 levels reached the very high levels observed at *Des*.

**Figure 6:**
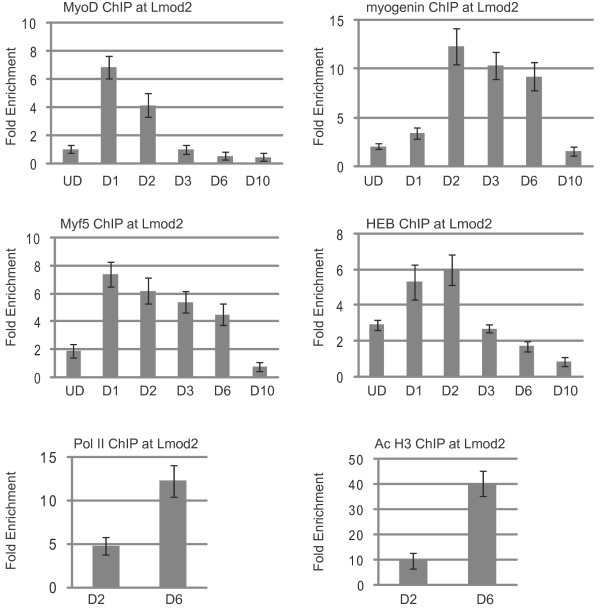
**Chromatin immunoprecipitation (ChIP) analysis of the *leiomodin 2 *(*Lmod2*) promoter**. Cross-linked extracts from proliferating myoblasts (UD, undifferentiated cells) and myofibers in differentiation media for the indicated number of days (D1-D10) were immunoprecipitated with antibodies against myogenin, MyoD, Myf5, HEB, RNA polymerase II (RNAP II), histone H3 acetylated at lysine 9 and/or 18 (H3 Ac9*/*18) or IgG. Immunoprecipitated DNA was purified and amplified with primers specific to the promoter of *Lmod2*. Relative enrichment at the *IgH *locus was used to normalize the data. The fold enrichment values were calculated as described in Methods.

The next promoter analyzed was the *Lmod2 *promoter (Figure 6). The expression of *Lmod2 *is dependent on myogenin during embryogenesis, and a promoter proximal binding site for myogenin has been defined [26]. A highly conserved noncoding region approximately 100 bp upstream of the predicted transcriptional start site contains two E boxes. The transcriptional start site of *Lmod2 *is immediately upstream of the translational start site, predicting a short 5' UTR of 112 bp. The sequence of the promoter proximal E box is CAGCTG, while the sequence of the promoter distal box E box is CAAATG. The E boxes are separated by 117 bp. Deletion of the promoter proximal E box in a luciferase reporter assay largely abolished the transactivation of the *Lmod2 *promoter by myogenin or MyoD [26]. At the *Lmod2 *promoter, we saw that HEB was present in proliferating cells, but none of the MRFs were significantly present. MyoD and Myf5 were recruited with a high affinity to the promoter on the first day of differentiation. Myogenin could be detected at this time point, but the binding ratio was relatively low. The association of myogenin increased greatly by two days of differentiation. Myogenin remained bound at six days of differentiation, but dissociated from the promoter by the tenth day of differentiation. MyoD and Myf5, recruited on the first day of differentiation, gradually dissociated from the promoter after this time point. MyoD was undetectable at three days of differentiation, while Myf5 remained associated with the promoter until sometime after six days of differentiation. The occupancy of HEB continued to increase during the initial stages of differentiation, peaking at two days of differentiation. HEB appeared to begin to dissociate from the promoter after two days of differentiation. HEB was only weakly detected at three days of differentiation and the levels continued to decrease at six days of differentiation, becoming undetectable by ten days of differentiation. The levels of RNAP II and AcH3 were significantly higher at six days of differentiation than at two days of differentiation.

**Figure 7:**
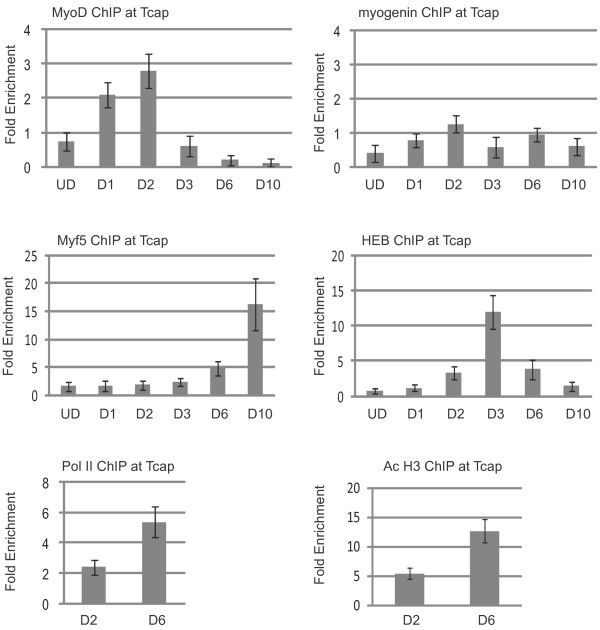
**Chromatin immunoprecipitation (ChIP) analysis of the *titin cap *(*Tcap*) promoter**. Cross-linked extracts from proliferating myoblasts (UD, undifferentiated cells) and myofibers in differentiation media for the indicated number of days (D1-D10) were immunoprecipitated with antibodies myogenin, MyoD, Myf5, HEB, RNA polymerase II (RNAP II), histone H3 acetylated at lysine 9 and/or 18 (H3 Ac9*/*18) or IgG. Immunoprecipitated DNA was purified and amplified with primers specific to the promoter of *Tcap*. Relative enrichment at the *IgH *locus was used to normalize the data. The fold enrichment values were calculated as described in Methods.

The final promoter analyzed was the *Tcap *promoter (Figure 7). The *Tcap *locus contains a highly conserved promoter proximal noncoding sequence that contains two E boxes. Interestingly, neither the promoter proximal E box (CATCTG) nor the promoter distal E box (CATGTG) is a favored binding site for MyoD or myogenin. However, MyoD and myogenin can activate this promoter, and myogenin binds to the promoter during embryogenesis [27]. In proliferating cells, none of the MRFs or HEB is bound. At day one of differentiation, MyoD was recruited to the *Tcap *promoter. MyoD remained associated with the promoter until two days of differentiation, but dissociated from the promoter by three days of differentiation. This result is consistent with our promoter characterization of *Tcap*, as we have shown that the promoter proximal E box is required for both the activity in C_2_C_12 _cells and the activation by MyoD in NIH3T3 cells [27]. Myogenin was not recruited to the *Tcap *promoter at any time point tested, which was surprising to us, as we have detected myogenin binding to the *Tcap *promoter in skeletal muscle tissue during embryogenesis [27]. The profile of Myf5 was particularly surprising. Myf5 was not associated with the promoter at early stages of differentiation, but could be weakly detected at the promoter after six days of differentiation. This binding increased greatly by ten days of differentiation. HEB could be detected on the *Tcap *promoter after two days of differentiation, when MyoD was still present. However, by day three, MyoD had departed the promoter and the occupancy of HEB increased greatly at this time. The peak of HEB binding appears to occur at three days of differentiation. By day six, HEB was at the same low level observed at two days of differentiation, and by ten days of differentiation it was undetectable. We found the pattern of MRF and HEB association at this gene particularly interesting, as there is almost no overlap between these factors. MyoD was recruited, followed by HEB, which then dissociated as Myf5 arrived. At this promoter, we again observed that RNAP II occupancy and H3 acetylation greatly increased at six days of differentiation as compared to two days of differentiation.

### HEB replaces E12/E47 at specific promoters during differentiation

**Figure 8:**
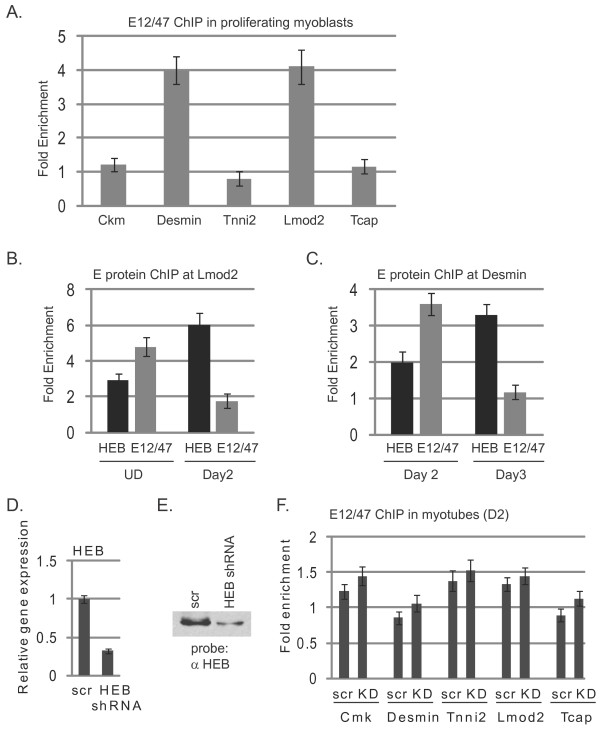
**E12/E47 and HEB exchange at the *leiomodin 2 *(*Lmod2*) and *desmin *(*Des*) promoters**. **(A) **E12/E47 binds to the promoters of *Des *and *Lmod2 *in myoblasts. Cross-linked extracts from proliferating myoblasts (UD, undifferentiated cells) were immunoprecipitated with antibodies against the *E2A *gene products. Immunoprecipitated DNA was purified and amplified with primers specific to the promoters of *Ckm*, *Des*, *Tnni2*, *Lmod2 *and *Tcap*. **(B) **E12/E47 and HEB exchange at the *Lmod2 *promoter. Cross-linked extracts from proliferating myoblasts and myofibers in differentiation media for two days were immunoprecipitated with antibodies against the *E2A *gene products, HEB or IgG. Immunoprecipitated DNA was purified and amplified with primers specific to the promoter of *Lmod2*. **(C) **E12/E47 and HEB exchange at the *Des *promoter. Cross-linked extracts from myofibers in differentiation media for two or three days were immunoprecipitated with antibodies against the *E2A *gene products, HEB or IgG. Immunoprecipitated DNA was purified and amplified with primers specific to the promoter of *Des*. Relative enrichment at the *IgH *locus was used to normalize the data. The fold enrichment values were calculated as described in Methods. **(D) **Gene expression analysis of *HEB *in cells expressing a small hairpin RNA (shRNA) construct targeting *HEB *or a scrambled control (scr). **(E) **Western blot analysis of the cells described in Figure 8D. The Western blot was probed with antibodies against *HEB*. **(F) **HEB is not required to displace E12/E47 at promoters. Results of chromatin immunoprecipitation assays performed after two days of differentiation on *HEB*-depleted cells and the scr control are shown.

At each of these promoters, we found the distinct pattern of HEB binding very surprising. We had anticipated seeing HEB bound to the promoter whenever an MRF was bound. While there are overlaps, the pattern of HEB binding is temporally controlled and unique to each gene. We wondered if perhaps these findings could be the result of the binding of the *E2A *gene products E12 or E47 in a compensatory fashion. We reasoned that E proteins might be associated at all time points, but the E protein could be either E12/E47 or HEB. To address this hypothesis, we repeated the ChIPassays on proliferating cells, on cells differentiated for two days and on cells differentiated for three days with antibodies against the *E2A *gene products. In proliferating cells, E12 or E47 could be detected at the *Des *and *Lmod2 *promoter binding sites (Figure 8A). For *Lmod2*, the binding of E12/E47 was detected with HEB in proliferating cells, but E12/E47 was lost as cells began to differentiate while HEB levels increased (Figure 8B). At two days of differentiation, E12/E47 could still be detected at the *Des *promoter, but that binding was lost at three days of differentiation (Figure 8C). At both of these promoter binding sites, we observed an exchange of E12/E47 and HEB as cells began to differentiate. E12/E47 was not detected on any of the other promoters assayed in this study. We have analyzed additional promoter proximal elements of differentiation-specific genes and have found that E12/E47 was associated with the *myogenin(myog)*, *troponin C, type 2(Tnnc2) *and *myosin heavy chain 3 *(*Myh3*) promoters in undifferentiated cells as well (Additional file 1 Figure S5A). We did not observe an association with the *troponin T, type 2 *(*Tnnt2*) promoter. In each case, binding was lost by two days of differentiation (data not shown). HEB was observed on the *Tnnt2*, *Myh3 *and *Tnnc2 *promoters following two days of differentiation (Additional file 1 Figure S5B). For the majority of the promoters analyzed here, it appears that HEB is the predominant E protein recruited during differentiation and that the transient association of HEB is not compensated by an overlapping pattern of E12/E47. These data are consistent with previously reported data that have established an important role for HEBβ in inducing differentiation [15]. Next, we asked whether the presence of HEB is required to displace E12/E47 from promoters as cells begin to differentiate. HEB levels were depleted with small hairpin (shRNA) constructs targeting HEB. Stable cell lines expressing these constructs were screened for expression of HEB and E12/E47. We proceeded with a construct that showed a 73% knockdown of HEB expression by RNA analysis (Figure 8D) and a reduction in protein expression by Western blot analysis (Figure 8E). No change in E12/E47 expression was detected by gene expression analysis (data not shown). ChIP assays with antibodies against E12/E47 were performed on cells differentiated for two days. We saw no enhanced association of E12/E47 in cells reduced in expression of HEB (Figure 8F), indicating that HEB is not required for the displacement of E12/E47 from promoters during differentiation.

### Myogenin, MyoD and Myf5 co-occupy promoters

**Figure 9:**
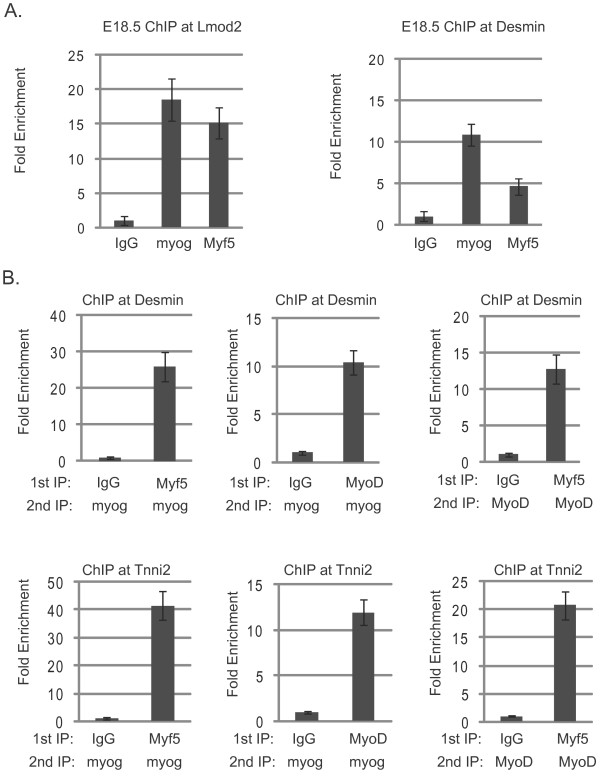
**Myogenin and Myf5 colocalize *in vivo *and co-occupy promoters with MyoD**. **(A) **Myf5 and myogenin colocalize on promoters during embryonic development. Chromatin immunoprecipitation (ChIP) analysis was performed on skeletal muscle from E18.5 embryos. The immunoprecipitated DNA was purified and amplified with primers specific to the promoter of *leiomodin 2 *(*Lmod2*) and *desmin *(*Des*). The fold enrichment values were calculated as described in Methods. **(B) **Myf5, myogenin and MyoD co-occupy promoters. Cross-linked extracts from myofibers in differentiation media for two days were immunoprecipitated with antibodies against the indicated antibody (1st IP) or IgG. The immunoprecipitated complexes were released and immunoprecipitated again with antibodies against the indicated antibody (2nd IP). The immunoprecipitated DNA was purified and amplified with primers specific to the promoters of *Tnni2 *and *Des*. Relative enrichment at the *IgH *locus was used to normalize the data. The fold enrichment values were calculated as described in Methods.

We were particularly interested in the binding profile of Myf5. For each gene tested, Myf5 had a unique binding pattern that was distinct from the binding patterns of MyoD and myogenin. We were surprised to observe that the profile of Myf5 overlapped with myogenin at several genes. To understand whether Myf5 and myogenin also colocalized during embryonic muscle development, we repeated the ChIP experiments in skeletal muscle tissue derived from embryonic day 18.5 (E18.5) embryos. We chose E18.5 as it is late in embryogenesis and is a time point at which myogenin is assumed to be highly active. Myf5 is known to function only during the early stages of myogenesis and thus would not be expected to contribute to gene expression at this stage. Moreover, Myf5 transcripts are not observed in embryos after E14 [28]. However, we detected Myf5 protein in hindlimb samples at late embryonic stages (Additional file 1 Figure S6). As expected, we detected myogenin at several muscle-specific promoters, including *Lmod2 *and *Des *(Figure 9A). Consistent with our C_2_C_12 _data, Myf5 colocalized with myogenin at both of these promoters *in vivo *(Figure 9A). After confirming that myogenin and Myf5 appear to bind to the same sequences at a late time point in embryogenesis, we were very interested in understanding whether the overlapping pattern was occurring in two cell populations or whether Myf5, myogenin and MyoD co-occupy promoters. To address this question, we performed sequential ChIP(ChIP-seq) assays for myogenin, MyoD and Myf5 in C_2_C_12 _cells. To address whether Myf5 and myogenin co-occupy promoters, cells were differentiated for three days, immunoprecipitated with Myf5 antibodies and subsequently immunoprecipitated with antibodies against myogenin. We detected co-occupancy of Myf5 and myogenin at the *Tnni2 *and *Des *promoters (Figure 9B). We also performed the experiment with antibodies against myogenin first, followed by immunoprecipitation with Myf5 antibodies, and, again, co-occupancy of Myf5 and myogenin on the *Tnni2 *and *Des *promoters was confirmed (data not shown). We next assayed for co-occupancy of MyoD and myogenin. Cells were differentiated for three days, immunoprecipitated with antibodies against MyoD and subsequently immunoprecipitated with antibodies against myogenin. Again, we observed co-occupancy of MyoD and myogenin on the *Des *and *Tnni2 *promoters (Figure 9B). Finally, we asked whether Myf5 and MyoD co-occupy promoters. Differentiated cell extract was immunoprecipitated with antibodies against Myf5 and subsequently immunoprecipitated with antibodies against MyoD. We observed that Myf5 and MyoD co-occupied the *Des *and *Tnni2 *promoters (Figure 9B). These data confirm that MyoD, myogenin and Myf5 are bound to the same promoter elements.

## Discussion

We have found that each muscle gene assayed showed a unique temporal association of the MRFs and E proteins. We were surprised to observe the dynamic and transient roles of the MRFs on each of these promoters. MyoD has been proposed to be a "pioneer" transcription factor required to initiate the cascade of regulatory events required to initiate expression of muscle-specific genes [29]. MyoD recruits chromatin-modifying activities that alter both the regional histone modifications and the chromatin remodeling at promoter binding sites [7,30]. It is thought that these events then allow the subsequent binding and transcriptional activity of myogenin. Our data are consistent with this model, as we observed early associations of MyoD followed by the association of myogenin. Our gene expression data also show that for most genes examined in this study, the recruitment of myogenin is coincident with high levels of transcription. Our data are consistent with those reported in other studies that showed that at the genes whose expression marks late myogenesis, *Ckm *and *Des*, MyoD is bound first, followed by the appearance of myogenin, Mef2 (myocyte enhancer factor 2) and Brg1 (Brahma-related gene 1), the catalytic subunit of the Swi/Snf chromatin remodeling complex [25]. In this prior study, it was also shown that the recruitment of myogenin was coincident with high levels of transcription of these genes in embryonic tissue. In our study, the peak of transcription and myogenin binding correlated with high levels of RNAP II promoter occupancy and histone H3 acetylation at the majority of genes assayed. We note that the histone H3 acetylation levels continued to rise following the departure of MyoD at several genes. These data suggest that while MyoD may be the initiating factor for chromatin modifications at the promoter, the continued presence of MyoD is not required for further increases in histone H3 acetylation.

The pattern of Myf5 binding was highly surprising. On certain genes, the Myf5 binding pattern overlapped with MyoD, but at other genes, the binding pattern overlapped with myogenin. In each case, the binding profile of Myf5 suggests that Myf5 has a previously uncharacterized role in mediating gene expression in differentiating cells. While it is known that Myf5 cannot mediate differentiation without myogenin or MyoD [8], our data suggest that Myf5 does cooperate with both MyoD and myogenin. Other groups have suggested that C_2_C_12 _cells, or the C_2 _cells used to derive them, have two populations of myoblasts: a MyoD-expressing population thought to be the differentiating population and a nondifferentiating or reserve population that expresses Myf5 [31,32]. However, in our studies, we can conclude that Myf5 is expressed in differentiating cells and that it colocalizes with MyoD and myogenin on specific promoters in C_2_C_12 _cells. Our data are highly suggestive that Myf5 plays a role in differentiation, but additional experiments are required to confirm this hypothesis.

The binding pattern of myogenin was surprising as well. The association of myogenin with muscle-specific genes as cells began to differentiate was expected, as myogenin is greatly upregulated at this time. However, the relatively brief association of myogenin with target genes was unexpected. A transient role of myogenin on target genes has previously been suggested, as myogenin appears to have distinct target gene sets during embryogenesis and in adult satellite cells and adult tissue [26,33]. Our data suggest that myogenin may mediate changes at the promoter that maintain high levels of expression without the continued presence of myogenin. Candidates for such a change include the switching of core promoter complexes, which has been observed in skeletal muscle differentiation. A TATA-binding protein (TBP)-related factor, TRF3, and an associated TBP-associated factor, TAF3, have been shown to be targeted by MyoD to the *myogenin *promoter following differentiation [31,34]. *TBP *is expressed in proliferating myoblasts, but following differentiation, *TBP *is downregulated and *TRF3 *and *TAF3 *are upregulated. It is also possible that myogenin may direct epigenetic changes that maintain gene expression.

The binding pattern of HEB was very surprising to us as well. Detailed biophysical experiments have shown that MRF and E protein heterodimers are highly stable when bound to DNA. These studies have also indicated that heterodimers likely form on the DNA. MyoD and E47 heterodimers are not detected in diluted conditions without DNA. However, in the presence of DNA, heterodimeric complexes are formed almost exclusively [35]. Additional work has shown that the weak MyoD homodimers and heterodimers that can form in the absence of DNA are equally stable [36]. This suggests that the MRFs and E proteins are likely to be monomeric in the cell. In this work {Maleki, 2002 #376}, it was also shown that while MyoD or myogenin E protein heterodimers on DNA were the most energetically favorable, MyoD and myogenin homodimers can bind E boxes with considerable positive cooperativity, while E12 homodimers exhibited negative cooperativity. The negative cooperativity of E12 suggests that the heterodimer may form on DNA by binding of the E12 monomer followed by binding of the MRF monomer.

Given these data, we anticipated detecting E proteins on the DNA throughout the time course of differentiation. Instead, we found a highly dynamic pattern of recruitment and release of HEB. This pattern was not compensated by E12/E47, as we observed E12/E47 binding to only two of the promoters in this study at early time points. At three days of differentiation, E12/E47 was not detected at any of the promoters analyzed. At *Des*, the only gene highly expressed during proliferation examined in this study, we did observe an association with both E12/E47 and HEB in proliferating cells. At *Lmod2*, we also observed an early recruitment of E12/E47 and HEB, whereas we observed only late recruitment of HEB at genes such as *Tnni2*. We hypothesize that E12/E47 might be required at a subset of genes whose expression is immediately required as cells begin to differentiate. While *Lmod2 *is not significantly expressed in proliferating cells, *Lmod2 *is upregulated very rapidly upon differentiation, and while the expression does continue to increase over an extended time course, the expression increases only two fold. This is in contrast to genes such as *Tnni2*, where the expression level increases ten fold over the extended time course. *Lmod2 *does not reach the high levels of transcription seen at *Tnni2 *that coincide with the peak of HEB binding. It is possible that the early recruitment of E12/E47 and HEB at *Lmod2 *helps to support a relatively constant level of expression that initiates immediately upon differentiation. It is striking that at both genes where we observed the binding of E12/E47, we also observed that HEB appeared to replace E12/E47 as cells began to differentiate. The binding pattern of HEB at *Tcap *is particularly interesting. HEB binding peaks at a time point when no MRFs are detected. Reduced levels of binding are detected at two additional time points when MyoD and Myf5 are bound on the individual days. Thus, while the HEB binding profile does overlap with MRF binding as predicted by the biophysical studies, the occupancy of HEB does not always overlap with the occupancy of the MRFs.

While these data have revealed many novel findings regarding the recruitment of the MRFs and E proteins, many questions remain. The additional factors and DNA elements that mediate the individual recruitment and release of each of these factors remain to be characterized. Many elegant studies of the role of chromatin modification in muscle differentiation have suggested that epigenetic events are important mediators in the activation of muscle genes. The Swi/Snf chromatin remodeling complex promotes muscle differentiation, and it is known that the Swi/Snf complex is recruited to both the *Des *and *Ckm *promoters studied here [25,37]. Important questions for future studies include how chromatin remodelers and chromatin-modifying enzymes contribute to the recruitment and release of the myogenic regulatory factors and E proteins to regulate muscle gene expression.

## Conclusions

Here we have shown that MyoD, myogenin and Myf5 have sequential and transient roles on each of the promoters assayed. For almost every gene assayed, we found that the binding of myogenin and HEB correlated with high levels of RNAP II occupancy, histone H3 acetylation and the peak of transcription as assayed by mRNA levels. We found that the primary E protein recruited to late differentiation genes is HEB. At the few promoters where E12/E47 was detected at early stages, HEB replaced E12/E47 during differentiation. Finally, we have shown that MyoD, myogenin and Myf5 colocalize on promoters, suggesting that Myf5 contributes to the gene expression of late differentiation genes.

## Methods

### Cell culture

Cells were grown in a humidified chamber at 37°C with 5% CO_2_. Proliferating C_2_C_12 _myoblasts (American Type Culture Collection, Manassas, VA, USA) were grown in Dulbecco's modified Eagle's medium (DMEM) supplemented with 10% fetal bovine serum (Thermo Scientific HyClone, Logan, UT USA. To induce differentiation into myotubes, cells were grown to 70% confluence and media were switched to DMEM supplemented with 2% horse serum (Thermo Scientific HyClone, Logan, UT USA). C_2_C_12 _cells were grown in differentiation medium for the number of days indicated in each experiment.

### Western blot analysis

Cell extracts were made by lysing phosphate-buffered saline-washed cell pellets in radioimmunoprecipitation assay buffer supplemented with protease inhibitors (Complete Protease Inhibitor Cocktail Tablets; Roche Applied Science, Indianapolis, IN USA. Following incubation on ice, clear lysates were obtained by performing centrifugation. Protein concentrations were determined by using the Bio-Rad Protein Assay (Bio-Rad, Hercules, CA USA. For each sample, 30 μg of protein were loaded onto each gel. Proteins were transferred onto a nitrocellulose membrane using a tank blotter (Bio-Rad, Hercules, CA USA), then blocked using 5% milk and 1× Tris-buffered saline plus Tween 20 (TBST) and incubated with primary antibody overnight at 4°C. Membranes were then washed with 1× TBST and incubated with the corresponding secondary antibody. Membranes were again washed with 1× TBST, incubated with chemiluminescence substrate according to the manufacturer's protocol (SuperSignal West Pico Chemiluminescent Substrate; Pierce Biotechnology, Rockford, IL USA and visualized by autoradiography. The antibodies used include anti-HEB (A-20; Santa Cruz Biotechnology, Santa Cruz, CA, USA), anti-E12/E47 (Yae; Santa Cruz Biotechnology), anti-Myf5 (C-20; Santa Cruz Biotechnology), anti-MyoD (5.8A; Santa Cruz Biotechnology), anti-GAPDH (anti-glyceraldehyde 3-phosphate dehydrogenase; Chemicon International, Billerica, MA USA) and anti-MyoG (F5D).. The F5D antibody developed by W. E. Wright was obtained from the Developmental Studies Hybridoma Bank under the auspices of the NICHD and maintained by the University of Iowa, Department of Biology, Iowa City, IA USA. Normal rabbit immunoglobulin G (IgG) (Santa Cruz Biotechnology, Santa Cruz, CA USA) was used as a nonspecific control.

### Quantitative reverse transcriptase-polymerase chain reaction assays

RNA was isolated from C_2_C_12 _cells by TRIzol reagent extraction (Invitrogen, Carlsbad, CA. Two micrograms of total RNA were reverse-transcribed with MultiScribe™ Reverse Transcriptase (Applied Biosystems, Carlsbad, CA USA. cDNA equivalent to 40 ng was used for quantitative reverse transcriptase-polymerase chain reaction (qRT-PCR) amplification (Applied Biosystems, Foster City, CA USA) with SYBR Green PCR Master Mix (Applied Biosystems, Foster City, CA USA). Samples in which no RT was added were included for each RNA sample. For measurements of relative gene expression, a fold change was calculated for each sample pair and normalized to the fold change observed at *HPRT*. mRNA expression levels were quantitated using a calibration curve based on known dilutions of concentrated cDNA. Each mRNA value was normalized to that of *HPRT*. Fold change was calculated by dividing the mRNA expression values of each sample pair. qRT-PCR data were calculated using the comparative *C*_t _method (Applied Biosystems, Foster City, CA USA). Standard deviations from the mean of the Δ*C*_t _values were calculated from three independent RNA samples and used to generate error bars. Intron-spanning primers to the coding region of *Lmod2*, *Des*, *Tnni2*, *Ckm *and *Tcap *are described in Additional file 1 Table S1. All qPCR assays were performed in triplicate, and at least two independent RNA samples were assayed for each time point.

### ChIP assays

Cell culture ChIP assays were performed and quantified as described previously [38] with the following modifications: 1 × 10^7 ^cells were used for each immunoprecipitation, and protein A agarose beads (Invitrogen) were used to immunoprecipitate the antibody-antigen complexes. ChIP assays of embryonic tissue were performed as previously described [26]. Limb tissue from wild-type C57BL/6 mice{Jackson Laboratory, Bar Harbor, ME USA) was isolated, cross-linked and enriched for nuclei. Nuclear extracts of limb tissue were precleared using incubation with protein A agarose beads (Invitrogen, Carlsbad, CA USA), which were also used to immunoprecipitate the antibody complexes from tissue extracts following antibody addition to the incubation mix. Antibodies against the following proteins were used: MyoD (5.8A; Santa Cruz Biotechnology, Santa Cruz, CA USA), HEB (A-20; Santa Cruz Biotechnology, Santa Cruz, CA USA), Myf5 (C-20; Santa Cruz Biotechnology, Santa Cruz, CA USA), E proteins (Yae; Santa Cruz Biotechnology, Santa Cruz, CA), myogenin (F5D; Developmental Studies Hybridoma Bank), RNAP II (H-224; Santa Cruz Biotechnology, Santa Cruz, CA USA) and histone H3 acetylated at lysine 9 and/or 18 (H3.Ac9*/*18; Millipore, Billerica, MA USAUpstate Biotechnology. Primers spanning the described promoter elements of *Lmod2*, *Des, Tnni2*, *Ckm*, *Tcap, Myog, Tnnt2, Myh3 and Tnnc2 *are described in Additional file 1 Table S1. 2^-[Δ][Δ]*C*t ^values were calculated using the following formula based on the comparative *C*_t _method: Δ*C*_t_, template (antibody) - Δ*C*_t_, template (IgG) = 2^-[Δ][Δ]*C*t^. Fold enrichments were determined using the formula: 2^-[Δ][Δ]Ct ^(experimental)/2^-[Δ][Δ]*C*t ^(reference, IgH). The standard error of the mean was calculated on the basis of replicate Δ*C*_t _values. The *immunoglobulin H (IgH) *locus was used to normalize the fold enrichments for the individual promoters. All ChIP assays shown in the figures are representative of at least three individual experiments.

### ChIP-seq assay

ChIP-seq analysis was performed as previously described [39] with antibodies against myogenin (F5D; Developmental Studies Hybridoma Bank, Iowa City, IA USA) and Myf5 (C-20; Santa Cruz Biotechnology, Santa Cruz, CA USA).

### shRNA knockdown

Cell lines depleted for HEB were constructed with shRNA constructs designed by the RNAi Consortium in the pLOK.1 plasmid (Open Biosystems, Huntsville, AL USA**)**. Five constructs targeting murine HEB and one scrambled control were linearized, transfected into C_2_C_12 _cells and selected with 2 μg/ml puromyosin. Pooled clones were selected and propagated. Depletion was confirmed at the RNA and protein levels. For the HEB depletions, the expression of E12/E47 was also confirmed at the RNA and protein levels.

## Competing interests

The authors declare that they have no competing interests.

## Authors' contributions

PL and JD designed the experimental approach. PL performed the described experiments, and JD wrote the manuscript with assistance from PL. Both authors read and approved the final manuscript.

## Supplementary Material

Additional file 1 **Davie Supplemental figures and Table SM**. Supplemental Figures S1 through S6, Table 1 and supplemental methods are included in this file. Click here for file
