# Supplementary material for: Sequential association of myogenic regulatory factors and E proteins at muscle-specific genes
Source: Skelet Muscle. 2011 Apr 4;1:14. doi: 10.1186/2044-5040-1-14 (PMC3156637; doi:10.1186/2044-5040-1-14)
Supplement: Additional file 1 — Davie Supplemental figures and Table SM. Supplemental Figures S1 through S6, Table 1 and supplemental methods are included in this file. [file 2044-5040-1-14-S1.PDF]

Supplemental Figure 1

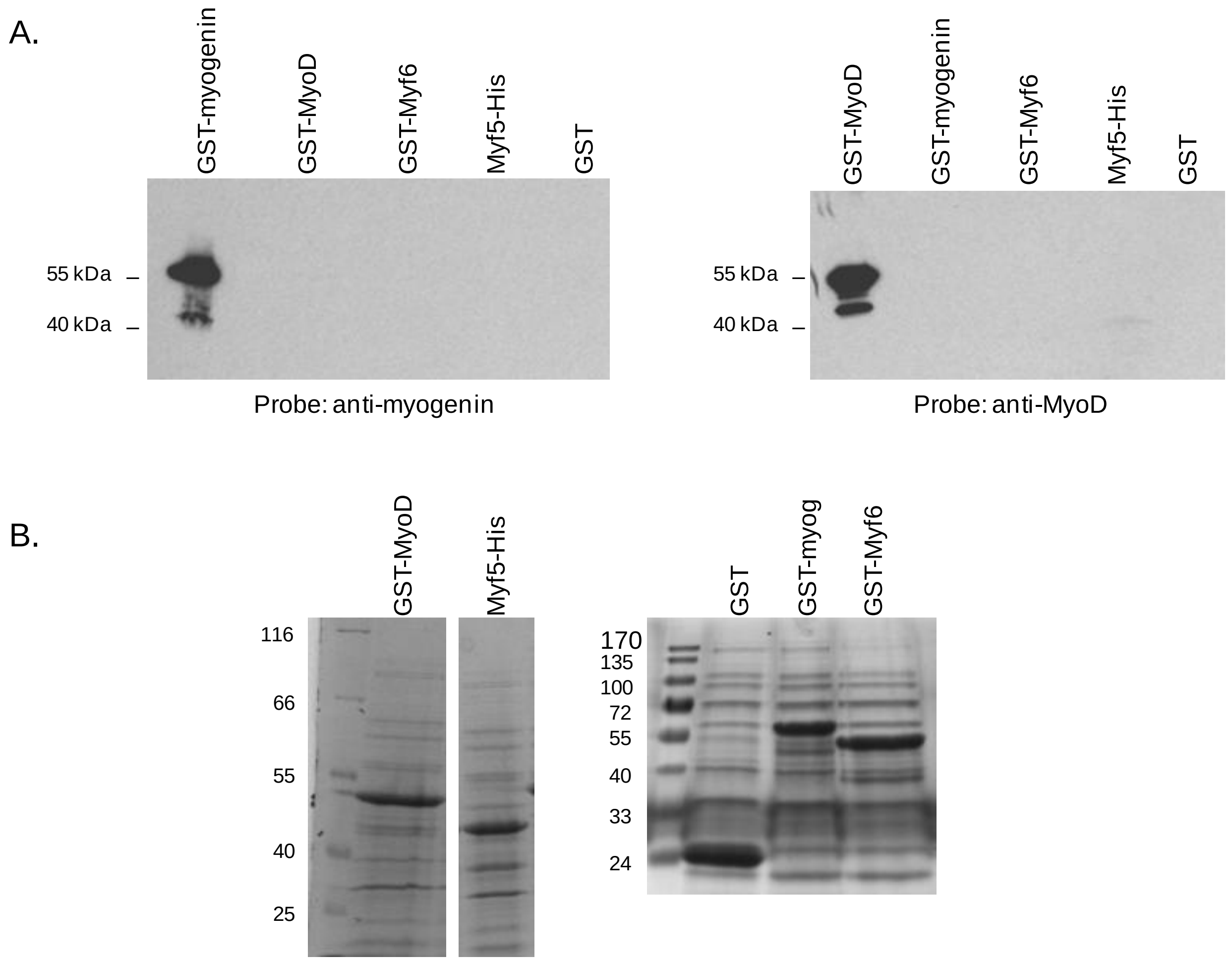

Supplemental Figure 1. Myogenin and MyoD antibodies specifically recognize target proteins.

A. Recombinant GST-myogenin, GST-MyoD, GST-Myf6 and Myf5-His were probed with antibodies against myogenin (F5D, Developmental Studies Hybridoma Bank) or MyoD (5.8A, Santa Cruz Biotechnologies) B. Expression of recombinant proteins. Each construct used in A. was induced with IPTG, and protein expression visualized by coomasie staining. For western blots, the recombinant proteins were loaded at 1/50 of the concentration shown in panel B.

Supplemental Figure 2

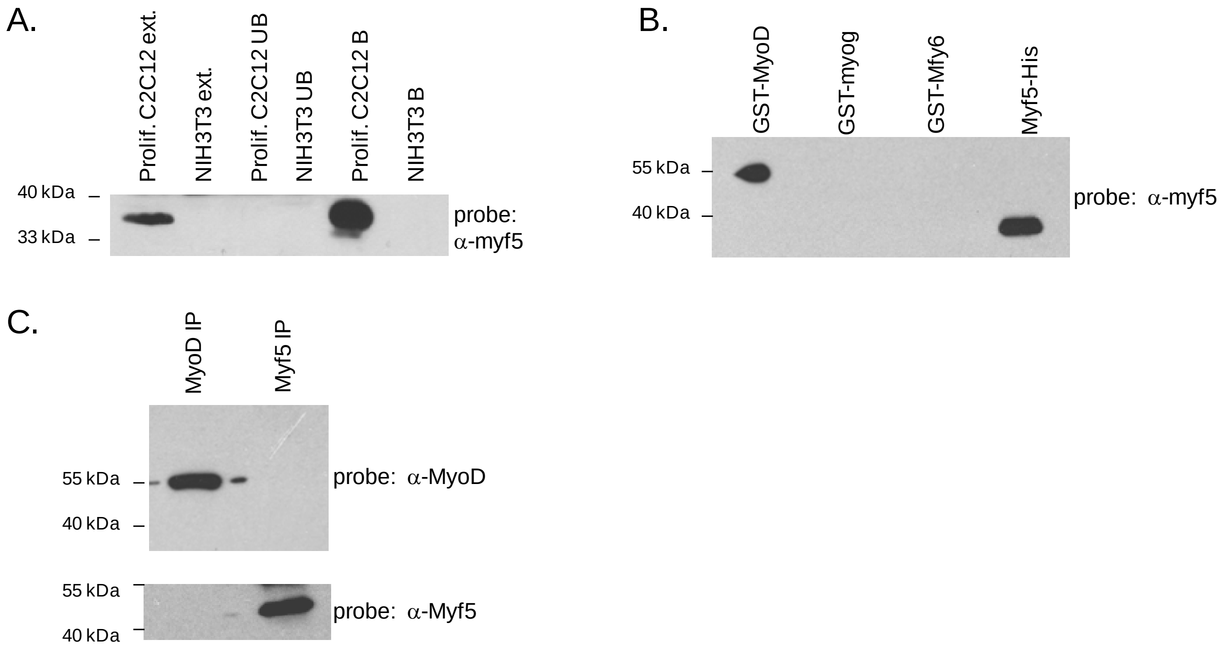

Supplemental Figure 2. Myf5 antibodies specifically immunoprecipitate Myf5.

A. Antibodies against Myf5 recognize and immunoprecipitate a band of the appropriate molecular weight in C2C12 cells. Extracts from proliferating C2C12 cells and NIH 3T3 cells, which do not express the MRFs, were immunoprecipitated with antibodies against Myf5 (C-20, Santa Cruz Biotechnologies). Immunoprecipitated samples are labeled B (bound fraction). The supernatant is labeled UB (unbound fraction). The western blot was probed against Myf5. B. The Myf5 antibody can detect recombinant MyoD, but not myogenin or Myf6. Recombinant GST-MyoD, GST-myogenin, GST-Myf6 and Myf5-His were probed with the Myf-5 antibody. C. The Myf5 antibody does not immunoprecipitate MyoD. Recombinant GST-MyoD and Myf5-His were immunoprecipitated with the Myf5 antibody and probed with antibodies against MyoD (upper panel). The blot was stripped and reprobed with antibodies against Myf5 (lower panel). For B.-C., expression of the recombinant proteins is shown in Supplemental Figure 1.

Supplemental Figure 3

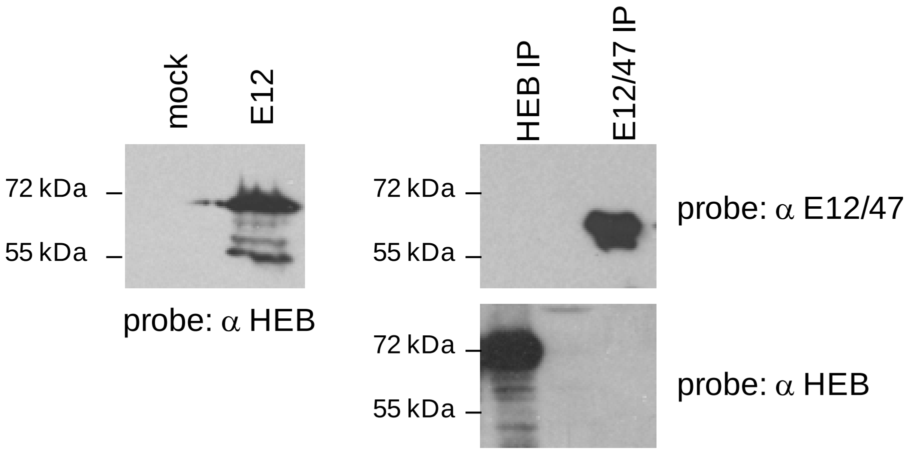

Supplemental Figure 3. Antibodies against HEB also recognize E12, but do not immunoprecipitate E12.

A. Antibodies against HEB recognize E12. HEK293 cells were transfected with E12-pCLBabe (Yang Z et al. Genes Dev. 2009 Mar 15. 23(6):694-707, Addgene plasmid 20918, or an empty vector (mock) and probed with antibodies against HEB (A-20, Santa Cruz Biotechnologies). B. Antibodies against HEB do not immunoprecipitate E12. Extract from HEK cells transfected with E12-pCLBabe were immunoprecipitated with antibodies against HEB (A-20, SCBT) or E12/47 (Yae, SCBT). The western blot was probed with antibodies against E12/47 (upper panel) and HEB (lower panel).

Supplemental Figure 4

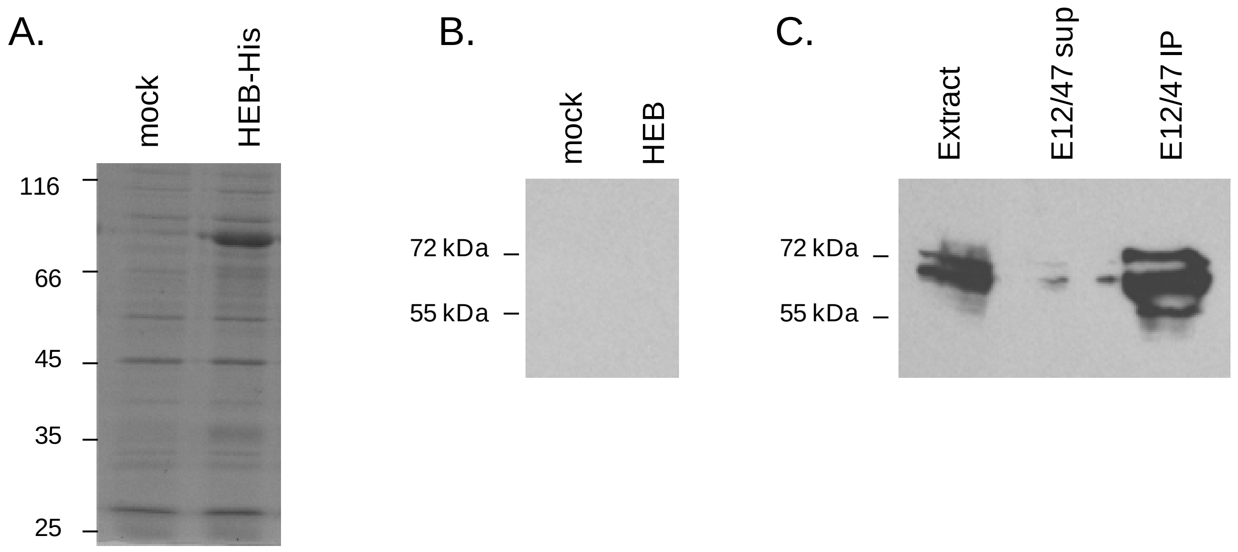

Supplemental Figure 4. Antibodies against E12/47 immunoprecipitate E12 and do not recognize HEB.

A. Expression of recombinant HEB. His tagged HEB was induced with IPTG and expression analyzed by commassie staining. B. Antibodies against E12/47 do not recognize HEB. The recombinant protein shown in A. was probed with antibodies against E12/47 (Yae, SCBT). The protein was used at the same concentration shown in A. C. Antibodies against E12/47 immunoprecipitate E12. Extract from HEK cells transfected with E12-pCLBabe were immunoprecipitated with antibodies against E12/47 (Yae, SCBT). Shown is the starting extract (Extract), the immunoprecipitated material (E12/47 IP) and the unbound fraction (E12/47 sup). The western blot was probed with antibodies against E12/47.

Supplemental Figure 5

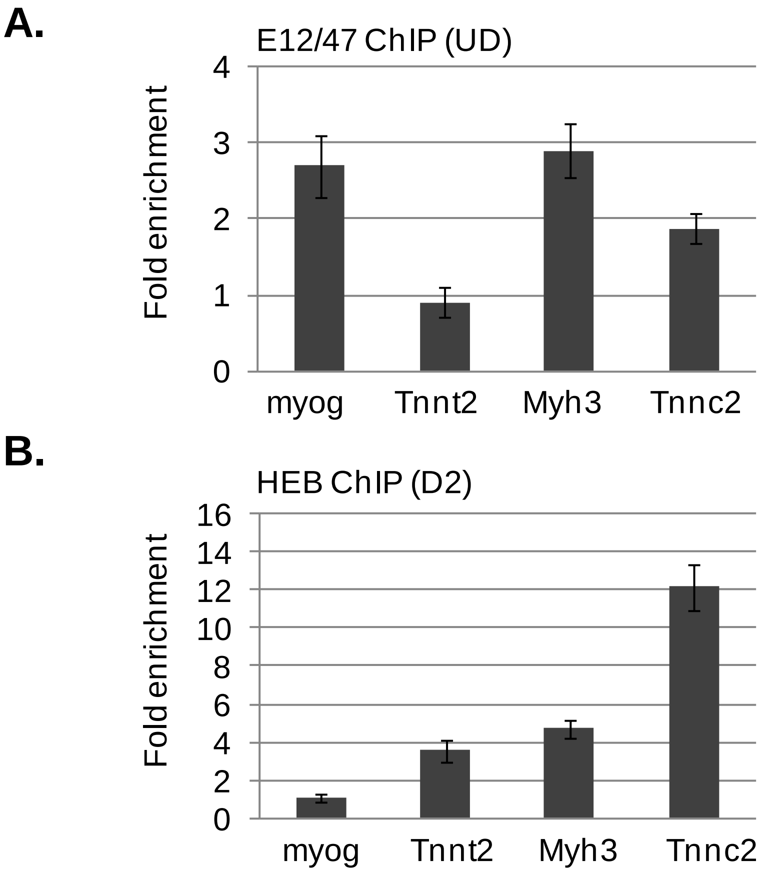

Supplemental Figure 5. Analysis of E12/47 and HEB occupancy at additional promoters.

A. E12/47 binds to the promoters of *myogenin* (*myog*), *myosin heavy chain 3* (*Myh3*) and *troponin C, type 2* (*Tnnc2*) in myoblasts. Binding to the *troponin T, type 2* (*Tnnt2*) promoter was not detected. Cross linked extracts from proliferating myoblasts (labeled UD) were immunoprecipitated with antibodies against E12/47 or IgG. Immunoprecipitated DNA was purified and amplified with primers specific to the indicated promoters. B. HEB occupies the *Tnnt2*, *Myh3* and *Tnnc2* promoters during differentiation. Binding to the myogenin promoter was not detected after two days of differentiation. Cross linked extracts from myofibers in differentiation media for two days were immunoprecipitated with antibodies against HEB or IgG. Immunoprecipitated DNA was purified and amplified with primers specific to the indicated promoters. Relative enrichments at the IgH locus were used to normalize the data. The fold enrichment values were calculated as described in Methods.

Supplemental Figure 6

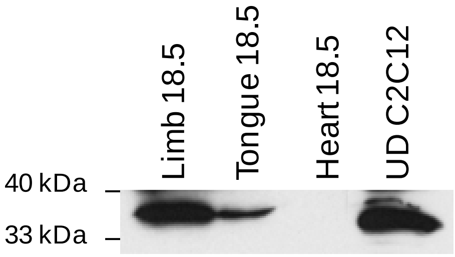

Supplemental Figure 6. **Myf5 is expressed at late embryonic stages.**

Extracts from hind limbs, tongue and heart from an E18.5 embryo and undifferentiated C2C12 cells were analyzed by western blot with antibodies against Myf5 (C-20, Santa Cruz Biotechnologies).

Supplemental Methods

Recombinant proteins

MyoD, myogenin and Myf6 were amplified from cDNA generated from RNA derived from embryonic limb skeletal muscle and cloned into pGEX-6P1 (GE Healthcare, Piscataway, NJ USA). Myf5 and HEB were amplified as above and cloned into pRSET A (Invitrogen, Carlsbad, CA USA). The construct E12-pCLBabe was obtained from Addgene courtesy of the Tapscott lab (Addgene plasmid 20918, Yang Z et al. Genes Dev. 2009 Mar 15. 23(6):694-707). The constructs were transformed into *E. coli* BL21 (DE3) (Stratagene, Santa Clara, CA USA). Single colonies were picked, grown at 37^0c to an OD_{600} of 0.4 and induced with 0.5mM IPTG. The cells were harvested 3 hours after induction and the supernatant fractions were collected as whole cell lysates.

Immunoprecipitations

For immunoprecipitations, transiently transfected HEK293 (ATCC) cells were harvested 48 h post transfection. Cells were scraped and lysed using radioimmunoprecipitation buffer (RIPA; 50mM Tris pH 8.0, 150mM NaCl, 0.1% SDS, 0.5% Deoxycholate, 1% NP40, 1mM EDTA) supplemented with protease inhibitors (Complete Protease Inhibitor, Roche Diagnostics, Indianapolis, IN USA). The cells were incubated on ice for 30 min with intermittent vortexing and extracts were obtained by centrifugation. Protein concentrations were determined using the Bio-Rad Protein Assay (Bio-Rad, Hercules, CA USA). 400-500 µg of the extract was incubated with either 2 µg of anti- MyoD (5.8A Santacruz Biotechnology (SCBT), Santa Cruz, CA USA), 2 µg of anti HEB(A20, SCBT) or 2 µg of anti E2A(Yae, SCBT) overnight at 4ºC with rotation. 20 µl of Protein A beads

(Invitrogen, Carlsbad, CA USA) were added the next day and samples were rotated at 4ºC for 2 hours. The beads were then washed 3X with RIPA buffer before adding 2X SDS loading buffer. Samples were boiled for 10 minutes and analyzed by Western blotting.

Transient Transfections

HEK293 cells (ATCC) were cultured in 10 cm plates 24 hours prior to transfection. Plasmids (10 µg) were transfected using calcium phosphate when the cells were 60% confluent. Cells were harvested 48 h post transfection.

Supplemental Table 1

Primers used for gene expression analysis:

Lmod2 F	5' ACCTTATCCCGATTTGCTGAAG 3'
Lmod2 R	5' ACCTTGAGCATGTCTGCAATG 3'
Desmin F	5' TTTCTCCACTCACAGGCTCTGA 3'
Desmin R	5' GCAGCATGAAGACCACAAAGG 3'
Ckm F	5' CCAGCCAGCCAGGGTCCCAA 3'
Ckm R	5' ACTCCTCATCGCCGGCCACA 3'
Tcap F	5' CCTTCTGGGCTGAGTGGAAA 3'
Tcap R	5' TCTGTGTATCCTCCTCGTGCAA 3'
Tnni2 F	5' GCCGCCGAGAATCTGAGA 3'
Tnni2 R	5' GACATGGAGCCTGGGATGTG 3'
HPRT1 F	5' TGACACTGGCAAAACAATGCA 3'
HPRT1R	5' GGTCCTTTTCACCAGCAAGCT 3'

Primers used for ChIP analysis:

Tnni2 E1,2F	5' GCCAAAGGAGCAAGAGTTAAAAAT 3'
Tnni2 E1,2 R	5' AGGAGAAAGTGTTCCCAAAATGTC 3'
Tcap E1 F	5' CCCATCACCACCAGTGAGTCT 3'
Tcap E1 R	5' GCCCTTTAAATAGCCCCTTCTTC 3'
Desmin P F	5' GTCTTCTGTCCTCTTGGGGCTGTCCA 3'
Desmin P R	5' TGGAGTGGATGTGAAGATGGGTGAC 3'
MCK P F	5' CGCCAGCTAGACTCAGCACT 3'
MCK P R	5' CCCTGCGAGCAGATGAGCTT 3'

Lmod2 E1U	5' CACCCTCTCCACATTGTCAC 3'
Lmod2 E1D	5' AAAGAACCAGGCATTCAAGG 3'
Tnnt2 E123 F	5' GGTGTTGGCTGCTATTTTGG 3'
Tnnt2 E123 R	5' GAATGTGCAGCAGGAGATG 3'
Tnnc2 E1 F	5' CCTAGAGTCCTAGCCCATTACC 3'
Tnnc2 E1 R	5' GAAACACATGTGCTGGAGTG 3'
myog E12 F	5' GGAATCACATGTAATCCACTGG 3'
myog E12 R	5' TCACACCAACTGCTGGGT 3'
Myh3 E12 F	5' CCTTTCTCTTCAGGCCACTAC 3'
Myh3 E12 R	5' TGACAGGGAGCTATGCCA 3'
IgH F	5' GCCGATCAGAACCAGAACACCTGC 3'
IgH R	5' TGGTGGGGCTGGACAGAGTGTTTC 3'
